# Book Review: Handbook of Positive Youth Development: Advancing Research, Policy, and Practice in Global Contexts

**DOI:** 10.3389/fpsyg.2021.746286

**Published:** 2021-10-25

**Authors:** Snežana Stupar-Rutenfrans, Aaron Johnson

**Affiliations:** University College Roosevelt, Middelburg, Netherlands

**Keywords:** positive youth development, methodological approaches, quantitative psychology, measurement, structural equation modeling

The *Handbook of Positive Youth Development: Advancing Research, Policy, and Practice in Global Contexts* edited by Radosveta Dimitrova and Nora Wiium brings together empirical research, methodological and theoretical perspectives, and implications for research, policy and practice. The volume includes 37 chapters that are innovative and diverse in theoretical and methodological approaches (e.g., cross-cultural, multi-national, experimental, longitudinal, mixed-methods) as well as inclusive with participants from diverse cultural, ethnic and sociodemographic backgrounds numbering a total of 22,083 youth and emerging adults as participants. The goal is to advance a multidisciplinary science scholarship on Positive Youth Development (PYD) toward a more inclusive understanding of the psychological experiences, mechanisms and correlates of positive development among young people in a global perspective.

The handbook sets a leading goal as that of tackling foundational problems and applications of statistical practice, global assessment and measurement. In so doing, the contributions cover a wide range of methods and practices in quantitative psychology and measurement such as best practices in quantitative analysis of multi-country comparisons and multi-groups data (e.g., developmental assets in India, Indonesia, Pakistan, Ghana, and Norway; risky behaviors in Colombia and Peru; healthy life styles in Mexico and mindfulness in Malaysia; academic achievement and risky behaviors among Albanians in Albania, Kosovo, Macedonia and Serbia; PYD measurement models in Bulgaria, Italy, Norway and Romania, China, Colombia, Italy, Jordan, Kenya, the Philippines, Sweden, Thailand, and the United States; PYD and violent radicalization in Canada etc.), culturally sensitive design of quantitative research and assessment across a variety of cultural groups, SEM perspectives in the application of quantitative methods to the study of human development through PYD approach, integration of quantitative methods, prevention and intervention research to mixed-methods designs and applications of these methods to policy, practice and program evaluation.

The volume is, thus, insightful for readers who are interested in methodological advances from the roots of PYD measurement and enlarging global comparative research in terms of advancement in measurement applications of youth development models (e.g., the 7Cs and the developmental assets models). Such applications reflect a major aim of this handbook particularly in under researched and neglected contexts from a global perspective. In so doing, the volume offers noteworthy progress and evidence in statistical procedures such as Structural Equation Modeling (SEM) and measurement invariance procedures embraced within new conceptual models of PYD, global measurement applications and practices of these models. Such procedures are embedded in newly developed measures on youth development with solid empirical testing of validity and reliability across cultural samples, regions and countries.

The handbook covers a wide range of health status topics across large, culturally, ethnically and socially diverse individuals and their level of wellness and illness, taking into account the presence of physical, mental, psychological and social well-being (e.g., injury, violence, nutrition and weight status, obesity, physical activity, healthy life behaviors, environmental quality, reproductive health, substance and alcohol use/abuse, domestic violence and abuse, self-harm and suicidal behaviors, maternal, child and infant health, access to health services, adolescent mental health, depression, anxiety etc.). The scope is to use PYD approach to promote healthy status among young people through health equity, thriving social and physical environments that promote good quality of life, healthy development, and healthy behaviors across the life span.

In brief, the handbook is insightful for new advancement in measurement applications, validity and reliability issues, structural equation modeling, measurement invariance across interdisciplinary academic areas as well as the nature of within and cross-country studies. For the concerned methodologist, it also demarcates a wide range of statistical testing techniques and provides an excellent framework of methodological applications for better science and scholarship from a variety of disciplines such as positive psychology, well-being studies, developmental psychology, child and family studies, cross-cultural psychology, education, prevention, intervention, intercultural relations, social psychology, sociology, methodology, counseling, community psychology, emerging adulthood and applied developmental science. Finally, its implications for practice must not be overlooked as the book will serve as a crucial resource to imbibe values related to diversity and a stepping stone to disseminate culturally sensitive counseling and patient care.

## Author Contributions

SS-R and AJ: writing and reviewing. Both authors contributed to the article and approved the submitted version.

## Conflict of Interest

The authors declare that the research was conducted in the absence of any commercial or financial relationships that could be construed as a potential conflict of interest.

## Publisher's Note

All claims expressed in this article are solely those of the authors and do not necessarily represent those of their affiliated organizations, or those of the publisher, the editors and the reviewers. Any product that may be evaluated in this article, or claim that may be made by its manufacturer, is not guaranteed or endorsed by the publisher.

